# Investigating the Significance of Non-*jejuni*/*coli Campylobacter* Strains in Patients with Diarrhea

**DOI:** 10.3390/healthcare11182562

**Published:** 2023-09-16

**Authors:** Nermin Teksoy, Mehmet Ilktac, Betigul Ongen

**Affiliations:** 1 Medical Microbiology Department, Istanbul Faculty of Medicine, Istanbul University, 34093 Istanbul, Turkey; nerminteksoy@gmail.com (N.T.); ongenb@gmail.com (B.O.); 2 Faculty of Pharmacy, Eastern Mediterranean University, via Mersin 10 Turkey, Famagusta 99628, Cyprus

**Keywords:** *Campylobacter* spp., *C. concisus*, gastroenteritis, multiplex PCR, non-*jejuni*/*coli*

## Abstract

*Campylobacter* is one of the most commonly reported foodborne bacteria worldwide. Although *Campylobacter jejuni* and *Campylobacter coli* have been reported to be responsible for the great majority of campylobacteriosis, the burden of infections by species other than *C. jejuni* and *C. coli* have been increasing as a result of a transition to diagnostic test methods that enable the isolation of emerging species. The aim of the present study was to recover *C. jejuni*, *C. coli*, and emerging species from the stool samples of 500 patients with gastroenteritis and 100 healthy subjects via the use of a filtration method and culture techniques using Butzler agar and mCCDA under a microaerobic or hydrogen-enriched atmosphere, identify the species by multiplex PCR methods and assess the significance of emerging species in enteric diseases. Thirty-one (6.2%) *Campylobacter* spp. were isolated from the stool samples of diarrheic patients but none from healthy individuals. Of 31 isolates, 21 (67.8%), nine (29%), and one (3.2%) were identified as *C. jejuni*, *C. coli*, and *Campylobacter concisus* by multiplex PCR, respectively. The filtration method was superior to the culture technique using mCCDA under a microaerobic atmosphere. *C. concisus* was evaluated as the etiology of gastroenteritis as a result of laboratory and clinical evaluations. The present study was the first to indicate that emerging *Campylobacter* species are rarely detected and *C. concisus* is linked to acute gastroenteritis in Turkey where additional studies are warranted to clarify the significance of emerging species in gastroenteritis.

## 1. Introduction

*Campylobacter* is a Gram-negative, spiral-shaped bacterium that is responsible for foodborne infections which are transmitted to humans mainly via the consumption of undercooked broiler meat. Campylobacteriosis was the most frequently reported bacterial foodborne infection in the United States of America in 2020 and the European Union (EU) in 2021, affecting 7208 and 127,840 individuals, respectively [1,2]. *Campylobacter* infections are also endemic with the pooled prevalence ranging from 8 to 10% in Africa and Asia [3,4,5].

*Campylobacter* infections are characterized by mild to moderate diarrhea that is generally self-limited. However, enteritis can be life-threatening due to severe dehydration especially among neonates, elderly individuals, and immunosuppressed patients. Campylobacteriosis is an important public health concern of global importance because of the post-infectious complications including Guillain-Barré syndrome, reactive arthritis, irritable bowel syndrome, and various systemic infections such as bacteremia, especially among the elderly and immunosuppressed individuals together with the concern for the treatment of the infections due to increased antibiotic resistance [6,7].

*Campylobacter jejuni* and *Campylobacter coli* have been reported to account for the great majority of the infections worldwide, although *Campylobacter fetus*, *Campylobacter upsaliensis*, and *Campylobacter lari* were also clearly linked to human infections [7,8]. However, the tendency for higher recovery rates of *C. jejuni* and *C. coli* can be due to bias that results from the preference of the diagnostic techniques (using antibiotic-containing selective media, a microaerobic atmosphere without H_2_ enrichment, incubation at 42 °C, and shorter incubation time) that are inappropriate for the detection of non-*jejuni*/*coli* species. By using a filtration method performed on non-specific media at 37 °C under a hydrogen-enriched microaerobic atmosphere or a PCR technique performed directly on fecal samples, non-*jejuni*/*coli* species were reported to account for as much as 50% of the *Campylobacter* species isolated from humans [9,10]. Although there has recently been an increase in the detection of emerging species due to improved culture media, growth conditions and implementation of molecular techniques, healthy subjects were not included in most of the studies that reported the detection of the strains from patients. Moreover, in a limited number of studies that investigated the presence of the strains both in patients with acute gastroenteritis and in a control group, the role of less commonly detected species, especially those in gastroenteritis that are human-hosted, such as *Campylobacter concisus,* was controversial. Thus, the role of *C. concisus*, if any, in gastroenteritis remains to be elucidated [11,12]. On the other hand, together with the implementation of molecular biology and sequencing techniques, accumulating data indicate that *C. concisus* may be associated with inflammatory bowel diseases such as active Crohn’s disease [12,13]. Increased scientific interest on emerging campylobacters whose relevance for public health is frequently underestimated will contribute to uncover the real burden of emerging species and a better understanding of the epidemiology of the infections.

*Campylobacter* spp. is one of the two most frequently detected bacteria among patients with acute gastroenteritis in Turkey [14]. Although *C. jejuni* and *C. coli* are the most frequent causal agents of campylobacteriosis, little is known about the occurrence of species other than *C. jejuni* and *C. coli* and their role in gastroenteritis in Turkey because culture methods used for the recovery of *Campylobacter* species are biased towards the detection of *C. jejuni* and *C. coli*.

The aim of this study was to investigate the presence of *Campylobacter* species both in patients with acute gastroenteritis and healthy individuals, compare different isolation techniques, identify the species using the multiplex PCR (mPCR) method, determine the utility of phenotypical tests in identification and assess the significance of emerging species in gastroenteritis.

## 2. Materials and Methods

### 2.1. Study Population

The sample size for the study was calculated by G*power version 3.1. At 95% confidence interval, with an effect size 0.112 and 80% power, at least 495 patients were required for inclusion into the study. Overall, 500 patients were included in the study between December 2016 and January 2018. Inclusion criterion for the patient selection was to have diarrhea. The patients with a history of antibiotic use in the last two weeks or who provided formed stool samples were excluded from the study. One hundred healthy individuals who did not consume antibiotics in the last two weeks were included in the study during the same period. Only one stool sample from each individual was tested. The research was approved by Istanbul University Istanbul Faculty of Medicine Ethics Committee (protocol code 2015/1246 and date of approval: 26 June 2015).

### 2.2. Campylobacter Isolation

#### 2.2.1. Culture Method

Stool samples were directly inoculated on two modified charcoal cefaperazone deoxycolate agar (mCCDA; Oxoid, Hampshire, UK) and a Butzler agar (Oxoid, Hampshire, UK). Butzler agar and one of the mCCDA were incubated at 37 °C for 72 h under the classical microaerobic atmosphere (5% O_2_, 10% CO_2_, 85% N_2_) generated using gaspak kits (Becton Dickinson, Sparks, MD, USA). The remaining mCCDA was incubated for six days at 37 °C under an H_2_-enriched microaerobic atmosphere consisting of a gas mixture of 1.5% O_2_, 7% H_2_, 10% CO_2_ and 81.5% N_2_ created by a gas replacement in a jar.

#### 2.2.2. Filtration Technique

Ten drops of a stool suspension prepared in sterile saline were deposited on a 0.6 µm pore-size membrane filter which was applied on the center of tryptose agar (Oxoid, Hampshire, UK) supplemented with 5% unlysed horse blood. Following a 15–20-min filtration process, the filter was removed under aseptic conditions and the medium was incubated at 37 °C under a microaerobic environment enriched with H_2_ for six days [9].

### 2.3. Identification of Campylobacter Species

#### 2.3.1. Phenotypical Identification

Following isolation by the culture method and/or filtration technique; presumptive colonies belonging to oxidase positive, L-alanine negative, Gram-negative bacteria with the characteristic gull-wing microscopical morphology were identified as *Campylobacter* spp. Strains that hydrolyzed hippurate were identified as *C. jejuni*. Catalase production, indoxyl acetate, pyrazinamide and urea hydrolysis, nitrate reduction, H_2_S production using a strip test and triple sugar iron (TSI) agar, ability to grow at 25 °C and 42 °C, in 1% glycine, on MacConkey agar, in the presence of a hydrogen-enriched atmosphere and cephalotin and nalidixic acid sensitivities of the strains were investigated [15,16,17].

#### 2.3.2. Molecular Identification

DNA of the *Campylobacter* strains which had been isolated on tryptose blood agar via the filtration technique were extracted using a commercially available DNA extraction kit (Invitrogen; PureLink, CA, USA). Genus-level identification of all isolates that were identified as *Campylobacter* spp. by phenotypical methods was confirmed by using C412F and C1288K primer pairs that were specific to the 16 S rRNA gene. PCR mixture consisted of 2.5 mM MgCl_2_, 200 μM dNTP, 0.4 μM primer pairs, 0.625 U Taq DNA polymerase, 1X Taq buffer, and 5 μL target DNA at the final volume of 50 μL. The amplification program was adjusted as follows: 94 °C 1 min, 55 °C 1 min and 72 °C 1 min for 25 cycles [18].

Species identification of all isolates that were confirmed as *Campylobacter* spp. by genus level PCR was performed by using mPCR test-1, -2 and -3. *C. jejuni*, *C. coli*, *C. lari*, and *C. upsaliensis* were identified by mPCR test-1 and *C. fetus, Campylobacter sputorum, Campylobacter curvus*, *Campylobacter helveticus*, and *Campylobacter mucosalis* by mPCR test-2. A 50 μL reaction mixture of both mPCR test-1 and mPCR test-2 included 2.5 U Taq DNA polymerase, 1× Taq buffer, 200 μm dNTP, 10 pmol/μL each of forward primers, 30 pmol/μL reverse primer and 3 μL target DNA [19]. The primers in the mPCR test-1 and the reverse primer in the mPCR test-2 were specific to *lpxA* as previously reported by Klena et al. [20]. The forward *lpxA* primers used in the mPCR test-2 was as follows: *C. fetus* (5′CGTTAGTTACCGTCCAGAAGAAAATACA3′, amplicon size: 162 bp), *C. sputorum* (5′TACTATTGGAGATGGCGGAAAAGTATTTAGC3′, amplicon size: 222 bp), *C. curvus* (5′GCAAGAGTCATCGGAAACACGCAAATA3′, amplicon size: 242 bp), *C. helveticus* (5′GACAAATTCATTCTAGTGCAGTGATT3′, amplicon size: 367 bp), and *C. mucosalis* [5′GTAGGCAAAAATGAGTAAAATTCATCATA3′, amplicon size: 381 base pairs (bp) [Miller W.G., personal communication]. The amplification programs of both of the tests were similar to the genus identification program except for the elongation step which was performed at 60 °C for 1 min [20]. Inconsistent results between phenotypical and mPCR tests for the identification of *C. jejuni* were confirmed by a PCR test using the primers Hip400F and Hip1134R which were specific to *hipO* gene as described previously [21].

For the differentiation of *C. concisus* and *C. mucosalis*, mPCR test-3 including Con1, Con2 and Muc1 primers that were specific to 23S rDNA was performed [22]. The reaction mixture at the final volume of 50 μL was as follows: 2.5 U Taq DNA polymerase, 1× PCR buffer, 200 μm dNTP, 5 pmol/μL of Con1 and Con2 primers each, 10 pmol/μL Muc1 primer, 1.2 mM MgCl_2_, and 3 μL target DNA. The amplification program was similar to that of mPCR test-1 and -2 [19,22]. The negative control that consisted of distilled water instead of target DNA and positive controls that included DNA extracted from the quality control strains were used for each run (Table 1). PCR products were visualized by ethidium bromide following gel electrophoresis using 1% and 2% agarose for genus and species identification, respectively.

### 2.4. Antibiotic Susceptibility Testing

Erythromycin, ciprofloxacin and tetracycline susceptibilities of the isolates that had been confirmed by PCR method were investigated by the disk diffusion method as suggested by the European Committee on Antimicrobial Susceptibility Testing (EUCAST) [23]. Because EUCAST did not provide clinical breakpoints of the antibiotics for non-*jejuni*/*coli* strains, antibiotic susceptibility testing of the strains was interpreted according to breakpoints provided for *C. jejuni* and *C. coli* strains.

**Figure 1:**
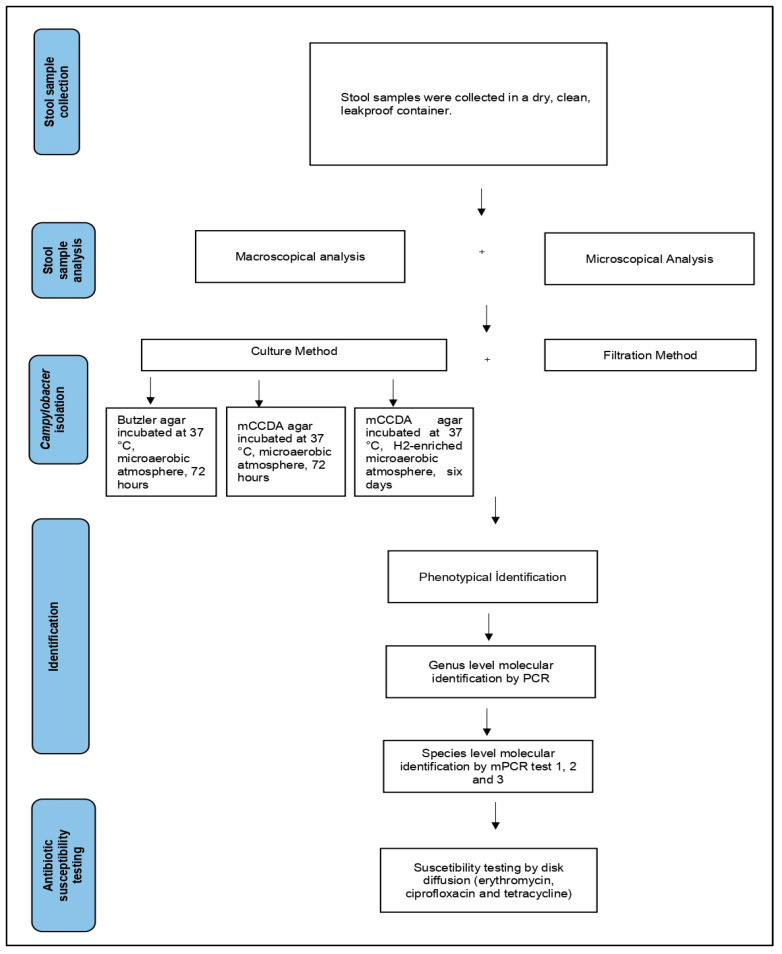
Flowchart of the experimental approach.

The experimental approach is shown as a flowchart in Figure 1.

### 2.5. Statistical Analysis

Statistical analysis of the data was carried out by Fischer’s exact test. A *p*-value less than 0.05 was considered as statistically significant.

## 3. Results

Of a total of 500 patients included in the study, 47.8% were female and 52.2% were male and 88 (17.6%) were 0–5, 64 (12.8%) were 6–17, 264 (52.8%) were 18–59, and 84 (16.8%) were ≥60 years old. Three hundred and eighty-nine were outpatients and 111 were inpatients. Macroscopical and microscopical analysis of the stool samples collected from the patients are shown in Table 2.

By using the different culture methods and filtration technique (Table 3), *Campylobacter* was isolated from 31 (6.2%) diarrheic patients of whom 18 were male and 13 were female. Of the 31 patients, 26 were outpatients and five were inpatients. PNLs were detected in 13 (42%) of the 31 patients. More than 70% (n: 22/31) of the stool samples collected from the patients who were infected with *Campylobacter* were either watery or watery with pus. No *Campylobacter* was detected in any of the healthy individuals.

Genus-level identification of all isolates that were identified as *Campylobacter* spp. by phenotypical methods was confirmed by PCR. Of the 31 strains, 21 (67.7%) were identified as *C. jejuni*, nine (29%) as *C. coli,* and one (3.2%) as *C. concisus* by mPCR tests.

The details of the isolation of *Campylobacter* species by the filtration technique, culture method using Butzler agar and mCCDA under both a microaerobic and hydrogen-enriched atmosphere are shown in Table 3. The filtration method was superior to the culture technique using mCCDA under a microaerobic atmosphere (*p* = 0.024), whereas no significant difference was found among other methods.

The oxidase test, indoxyl acetate hydrolysis, nitrate reduction, H_2_S strip test, growth at 42 °C and in 1% glycine were positive whereas H_2_S production on TSI, growth at 25 °C and on MacConkey agar were negative for all of the strains. The hippurate hydrolysis test was found to be positive and negative for all of the *C. jejuni* and *C. coli* strains, respectively. *C. concisus* hydrolyzed hippurate but was negative for the *hipO* gene. The catalase test was positive in all of the *C. jejuni* and *C. coli* isolates but negative in the *C. concisus* strain. The pyrazinamidase test revealed variable results for *C. jejuni* and *C. coli* strains. All of the *C. jejuni* and *C. concisus* strains were resistant to cephalotin whereas only one of the *C. coli* strains was susceptible to the antibiotic. All *C. coli* and *C. concisus* strains and 16 of 21 *C. jejuni* isolates were resistant to nalidixic acid (Table 4).

Of the 31 patients, nine were 0–5, six were 6–17, and 16 were >18 years old. Eighteen of them were female and 13 were male. Twenty-six patients were outpatients whereas only five were hospitalized. In addition to diarrhea, fever, abdominal cramp, and vomiting were among the most frequently detected clinical symptoms of the patients who were found to be positive for *Campylobacter* spp. Eleven of 31 patients had various comorbidities including Crisponi syndrome, renal failure, kidney transplantation, inflammatory bowel syndrome, chronic lymphocytic leukemia, Bartter syndrome, myeloma, cholecystectomy, diabetes, hypertension, cardiac dysrhythmia, and hypothyroidy (Table 5).

Of the 31 isolates, 29 (93.5%), 17 (54.8%) and one (3.2%) were resistant to ciprofloxacin, tetracycline and erythromycin, respectively. Of 21 *C. jejuni* strains, 19, 13 and one were resistant to ciprofloxacin, tetracycline and erythromycin, respectively. All *C. coli* strains were resistant to ciprofloxacin and susceptible to erythromycin. Tetracycline resistance was detected in three *C. coli* isolates. The *C. concisus* strain was resistant to ciprofloxacin and tetracycline but sensitive to erythromycin (Table 6).

### Clinical Features and Laboratory Findings of the Patient from Whom C. concisus Was Isolated

*C. concisus* was isolated from a 61-year-old woman. The patient had laparoscopic colesystectomy, diabetes, intermittent constipation, hypertension, cardiac dysrhythmia, and hypothyroidy. She presented to the emergency unit with complaints of vomiting, fatigue, abdominal cramp, and bloody diarrhea three times a day. Serum C-reactive protein (CRP) level was elevated. PNLs were detected in microscopical analysis of the loose stool sample collected from the patient. *Salmonella*, *Shigella*, *Aeromonas*, *Plesiomonas*, *Yersinia*, and *Vibrio* spp. were not detected in the routine stool culture. The patient was discharged from the hospital after the initiation of empirical metronidazole and ciprofloxacin treatment.

## 4. Discussion

Campylobacteriosis is one of the most frequently detected bacterial foodborne infections worldwide. Two thermotolerant species, *C. jejuni* and *C. coli*, have been reported to be responsible for the vast majority of the infections [8]. However, culture methods and incubation conditions generally used by the laboratories for the isolation of thermotolerant species do not support the isolation of non-thermotolerant strains that are often susceptible to antibiotics included in the selective media and require prolonged incubation under a hydrogen-enriched atmosphere. Thus, the occurrence of non-*jejuni*/*coli* strains of human origin is not well-recognized or underestimated. Lastovica et al. [9,24] recommended the Cape Town protocol based on the filtration technique using a non-selective medium and a hydrogen-enriched atmospheric environment as an alternative to the conventional culture method. The protocol was found to increase the isolation rate of *Campylobacter* spp. by three-fold compared to the direct culture on selective media [9]. The filtration technique was reported to be as efficient as culture method using mCCDA and more appropriate for the screening of all *Campylobacter* species and *Campylobacter*-like bacteria such as *Arcobacter* spp. [25]. In the present study, all of the 31 *Campylobacter* strains that were recovered from 500 patients were isolated by using the Cape Town protocol. Of 31 strains, 30 were recovered by traditional culture on Butzler agar under a microaerobic atmosphere, whereas 26 and 25 of the strains were detected on the mCCDA under hydrogen-enriched and microaerobic atmospheric conditions, respectively. The filtration method was found to significantly increase (*p* = 0.024) the isolation rate of *Campylobacter* spp. by 1.2-fold compared to the traditional culture method using mCCDA under a microaerobic atmosphere, whereas the recovery rates of *Campylobacter* by the Cape Town protocol and the conventional culture method using either Butzler agar under a microaerobic atmosphere or mCCDA under a hydrogen-enriched atmosphere were similar.

In a study carried out in South Africa using the Cape Town protocol, *C. jejuni* was the most frequently (32.3%) isolated species followed by *C. concisus* (25%) and *C. upsaliensis* (24%). Interestingly, species other than *C. jejuni* and *C. coli* accounted for nearly half of the *Campylobacter* species [9]. Vandenberg et al. [26] reported an increased recovery of *C. upsaliensis*, *C. concisus* and various other emerging species using the filtration method and incubating the media under a hydrogen-enriched atmosphere. In another study that was carried out in Denmark including 11,314 stool samples collected from patients with diarrhea, *C. concisus* (3.9%) was isolated as high as *C. jejuni* and *C. coli* (4.8%) via the filtration technique [27]. *C. jejuni* was the most frequently recovered species in the present study followed by *C. coli*. The two species were responsible for the great majority (96.7%) of the *Campylobacter* infections. Only one of the 31 strains was non-*jejuni*/*coli*, representing 0.2% of the patients and 3.2% of the *Campylobacter* species. The isolation rate (0.2%) of emerging *Campylobacter* species detected in our study is similar to that of another study performed by Vanderberg et al. [26] in Belgium. However, the rate of detection is lower than that reported in other studies carried out in South Africa, Belgium, Denmark, Sweden, and Canada [24,25,26,27,28,29,30]. The distribution of species found in this study that revealed the predominance of *C. jejuni* and *C. coli* is similar to the previous findings of our laboratory using classical culture techniques and incubation conditions that were optimal for the growth of thermotolerant species [31]. Moreover, the overall isolation rate of *Campylobacter* spp. that was 6.2% in this study is in parallel with that of another study in which the prevalence was 5.4% [32]. Thus, the present study indicates that non-*jejuni*/*coli* species are rarely detected and represent the minority of *Campylobacter* species.

Because of the long turnaround time of the culture method and fastidious nature of *Campylobacter* species, the PCR technique has recently been attractive for the investigation of *Campylobacter* spp. in stool samples. Bullman et al. [33] investigated the presence of *Campylobacter* spp. in the fecal samples of patients with diarrhea using the culture technique and molecular methods. Together with the overall increase in the detection of the *Campylobacter* species, less frequently isolated species such as *C. fetus*, *C. upsaliensis*, *C. lari*, and *Campylobacter hyointestinalis* represented 10% of the samples that were negative by culture. The authors reported *Campylobacter ureolyticus* as the second most frequently (41%) detected species following *C. jejuni* (51%).

Partly as a result of implementation of highly sensitive molecular techniques in the diagnosis, the role of emerging *Campylobacter* species in gastrointestinal diseases has been controversial because the species have also been detected in healthy individuals [12]. In a study that investigated the distribution of *Campylobacter*, *Helicobacter*, and *Arcobacter* species in the stool samples of healthy volunteers and patients with diarrhea, *C. concisus* and various non-*jejuni*/*coli* species, including *C. ureolyticus, Campylobacter hominis*, and *Campylobacter gracilis* were reported not to be associated with acute gastroenteritis because of the failure to show any significant difference in detection between patients and the control group [34]. Similar findings were also reported by Inglis et al. [29], Tilmanne et al. [35], and Van Etterijck et al. [28]. On the other hand, Collado et al. [36] reported a significant difference in the prevalence of *C. jejuni* and *C. concisus* between patients with acute gastroenteritis and healthy individuals, a finding that supported the role of the two species in intestinal disease. Similar findings supporting the role of *C. concisus,* a human-hosted species of which the primary colonization site is oral cavity, in gastroenteritis were reported in various studies as a result of failure to recover the species in healthy volunteers, ruling out the infections by the most frequently detected enteropathogens or taking into consideration the clinical findings [27,37,38]. Data supporting the role of *C. concisus* in gastrointestinal diseases such as inflammatory bowel disease have recently been accumulating via whole genome sequencing studies that revealed variations in the genomic content, affecting the pathogenic potential of the strains isolated from healthy subjects and patients with gastrointestinal system disease [13,39]. In the present study, *C. concisus* was recovered from the stool sample of a 61-year-old patient who was admitted to the emergency department with complaints of bloody diarrhea three times a day. Clinical findings, laboratory test results (increased serum CRP level, presence of fecal polymorphonuclear leukocytes), ruling out infections by the most frequently isolated bacterial acute gastroenteritis agents such as *Salmonella*, *Shigella*, *Aeromonas*, *Plesiomonas*, *Yersinia*, and *Vibrio* spp. and the detection of the agent only among the patient group were evaluated as indications that *C. concisus* was the etiology of diarrhea. Likewise, routinely investigated bacterial enteropathogens were negative in the stool cultures of the remaining 30 patients with clinical symptoms (Table 5) from whom *C. jejuni* or *C. coli* were detected as single causative agent.

In routine clinical microbiology laboratories, genus and species identification of *Campylobacter* species are generally carried out using phenotypic methods. In our study, a wide variety of biochemical tests were performed to investigate the utility of phenotypic tests for species identification. The hippurate hydrolysis test, one of the most frequently used tests that discriminates *C. jejuni* from other species, yielded a false-positive result for the *C. concisus* strain. In addition to false-positive hippurate test results for the strains that were reliably identified as species other than *C. jejuni* by molecular tests, false-negative results were also reported that brought the reliability of the test into question, especially for epidemiological investigations that are critical to intervene appropriate preventive strategies [31,32]. Nalidixic acid susceptibility testing was one of the historical phenotypical methods that was used for species-level identification of *Campylobacter* species. However, taking into account the high rate (26 of 31 strains) of resistance among *Campylobacter* spp., it is thought that the method has lost its significance in species identification, especially in regions where quinolone resistance is high. Similarly, indoxyl acetate and pyrazinamidase tests and the ability to grow on MacConkey agar yielded incompatible results with the generally accepted biochemical profiles of *C. jejuni*, *C. coli*, and *C. concisus*. Because phenotypic identification is challenging due to the biochemically inactive nature of *Campylobacter* species and due to the lack of definitive tests, the use of molecular techniques is recommended for surveillance and risk assessment studies that require prompt and accurate species-level identification.

An increase in the antibiotic resistance among *Campylobacter* spp. is an urgent global public health threat. In 2021, the average level of ciprofloxacin resistance in the EU was reported at high levels in *C. jejuni* (64.5%) and *C. coli* (69.6%) human isolates. Tetracycline resistance was high (45.3%) in *C. jejuni* and extremely high (70.3%) in *C. coli,* whereas erythromycin resistance was very low (1.1%) and low (8.5%) in *C. jejuni* and *C. coli*, respectively [40]. According to the National Antimicrobial Resistance Monitoring System (NARMS) 2018 data, the occurrence of ciprofloxacin, tetracycline and erythromycin resistance was 28.8%, 42.2%, and 2% in *C. jejuni* and 40.5%, 60.1%, and 13.3% *in C. coli* isolates of human origin in the USA, respectively [41]. In a recent study carried out in South Africa, erythromycin resistance of campylobacters isolated from pediatric stool samples was reported at a higher rate (24.4% in *C. jejuni* and 37.2% in *C. coli* isolates) whereas resistance to ciprofloxacin (16.6% in *C. jejuni* and 16.9% in *C. coli*) and tetracycline (28% and 18.6% for *C. jejuni* and *C. coli*, respectively) were found to be lower when compared to the EU and the USA [42]. Ciprofloxacin resistance was reported to be extremely high (over 70%), whereas tetracycline and erythromycin resistance were detected as 25% and 5.9% in Turkey, respectively [43,44]. In the present study, the rate of erythromycin resistance (3.2%) was lower whereas the rates of ciprofloxacin and tetracycline resistance were higher (93.5% and 54.8%, respectively) than those in the previous studies conducted in Turkey, highlighting the increase in the resistance rates of the latter two antibiotics in Turkey (Table 6).

Strengths of the present study include employing different isolation methods and incubation conditions for detection, comparing phenotypical and molecular techniques by applying a wide range of biochemical tests for identification, and indicating the potential contribution of *C. concisus* to acute gastroenteritis. On the other hand, there were two major limitations in this study that could be addressed in future research. One of the limitations included challenges in the isolation of *Campylobacter* species because cultivation techniques has been reported to have lower sensitivity than PCR-based culture independent diagnostic tests (CIDT) which detect the microorganisms directly in the stool sample. However, culture is still regarded as the golden standard method for the detection of *Campylobacter* spp., not only because questions have been raised regarding the clinical relevance of CIDT positive test results, but also these tests do not yield the bacterial isolates necessary for antibiotic susceptibility testing or essential public health surveillance activities such as molecular typing [45]. The second limitation is the selection bias where the patients were included in the study without considering their underlying diseases. Comorbidities such as immunodeficiencies including human immunodeficiency virus (HIV) infection were reported to be risks factor for campylobacteriosis [6]. Moreover, increased incidence of enteric *Campylobacter* infections have been revealed in men who have sex with men or individuals with high-risk sexual practices [46]. Thus, the comorbidities detected in some patients in the present study may affect the outcome of the study. Investigating the presence of emerging *Campylobacter* species among patients with similar comorbidities is recommended in future research.

## 5. Conclusions

The recent increase in the discovery of novel species alongside the rise in accumulating data that link emerging species to infections or intestinal diseases have brought some difficulties into routine diagnosis and species-level identification of clinical *Campylobacter* strains. Routine culture techniques and phenotypic identification methods used in the diagnostic laboratories may remain inefficient for the diagnosis and identification of species other than *C. jejuni* and *C. coli*. Nevertheless, epidemiological investigation by the use of a culture method that supports the recovery of emerging species in complement with molecular diagnostic techniques will contribute to the understanding of their roles in enteric diseases. The present study that included 500 patients with gastroenteritis and 100 healthy subjects is the first to investigate emerging *Campylobacter* species in Turkey. The study highlights the importance of using alternative culture methods and incubation conditions for isolation, and molecular techniques for identification of *Campylobacter* species. The study is the first to link *C. concisus* to acute gastroenteritis in Turkey. Further studies focusing on non-*jejuni*/*coli* species of *Campylobacter* are warranted to clarify the significance of these lesser-known species in intestinal diseases including gastroenteritis. The findings of this study are thought to provide valuable insights to the field.

## Figures and Tables

**Table 1:** Quality control strains included in the study.

Quality Control Strain	Collection Number
*C. jejuni*	NCTC 1168 (RM1862)
*C. coli*	RM2228
*C. upsaliensis*	RM3195
*C. lari*	RM2100
*C. fetus*	82-40 (RM15492)
*C. curvus*	525.92 (RM4077)
*C. helveticus*	ATCC 51209 (RM3228)
*C. concisus*	13826 (RM5485)
*C. mucosalis*	ATCC 43264 (RM4114)
*C. sputorum*	CCUG 20703 (RM4121)

**Table 2:** Distribution of the patients according to gender, hospital status, macroscopical and microscopical analysis of the stool samples [n (%)].

Gender ^1^		Hospital Status ^2^		Macroscopical Analysis of the Stool Samples ^3^							Microscopical Analysis of the Stool Samples ^4^	
F	M	O	I	W	WP	L	LP	WBP	WB	LB	PNL (+)	PNL (−)
239 (47.8)	261 (52.2)	389 (77.8)	111 (22.2)	175 (35)	173 (34.6)	119 (23.8)	18 (3.6)	11 (2.2)	3 (0.6)	1 (0.2)	168 (33.6)	332 (66.4)

^1^ F: Female, M: Male; ^2^ O: Outpatient, I: Inpatient; ^3^ W: Watery; WP: Watery with pus; L: Loose; LP: Loose with pus; WBP: Watery with blood and pus; WB: Watery with blood; LB: Loose with blood; ^4^ PNL (+): Polymorphonuclear leukocytes detected; PNL (−): Polymorphonuclear leukocytes not detected.

**Table 3:** Recovery of *Campylobacter* species by the filtration technique and culture methods using different media and incubation atmospheres (n).

	Filtration	Butzler Agar ^1^	mCCDA ^2^	mCCDA ^1^
*C. jejuni* (n: 21)	21	20	18	18
*C. coli* (n: 9)	9	9	7	6
*C. concisus* (n: 1)	1	1	1	1
Total (n: 31)	31	30	26	25

^1^ Incubated under a microaerobic atmosphere; ^2^ incubated under a hydrogen-enriched atmosphere.

**Table 4:** Positive test results of biochemical characteristics according to the species (n).

Species	Oxidase	Catalase	Hippurate	Indoxyl Acetate	Pyrazi-namidase	Nitrate	H_2_S Strip	Urease	H_2_S TSI	25 °C	42 °C	1% Glycine	Amino-peptidase	Mac-Conkey	Cephalotin (R) ^1^	Nalidixic Acid (R) ^1^
*C. jejuni* (n: 21)	21	21	21	21	20	21	21	0	0	0	21	21	0	0	21	16
*C. coli* (n: 9)	9	9	0	9	7	9	9	0	0	0	9	9	0	0	8	9
*C. concisus* (n: 1)	1	0	1	1	1	1	1	0	0	0	1	1	0	0	1	1

^1^ R: Resistant.

**Table 5:** Demographic and clinical characteristics of the 31 patients from whom *Campylobacter* spp. was isolated.

Patient No.	Age (years)	Gender ^2^	Hospital Status ^3^	Clinical Symptoms	Comorbidity	*Campylobacter* spp.
1	9 m ^1^	F	O	Diarrhea, fever	Crisponi syndrome	*C. jejuni*
2	11	M	O	Diarrhea, fever	-	*C. coli*
3	34	F	O	Diarrhea	-	*C. coli*
4	10	F	O	Diarrhea, abdominal cramp	-	*C. jejuni*
5	8	F	O	Diarrhea	Renal failure	*C. coli*
6	28	F	I	Diarrhea	Kidney transplantation	*C. jejuni*
7	1	M	O	Diarrhea	-	*C. jejuni*
8	1	M	O	Diarrhea, fever, vomiting	-	*C. jejuni*
9	37	M	I	Diarrhea, fever	Kidney transplantation	*C. coli*
10	8	F	O	Diarrhea, abdominal cramp	-	*C. coli*
11	2	M	O	Bloody diarrhea	-	*C. jejuni*
12	46	M	O	Diarrhea	IBS ^4^	*C. jejuni*
13	22	F	O	Diarrhea	-	*C. jejuni*
14	19	F	O	Diarrhea, fever	Kidney transplantation	*C. jejuni*
15	57	M	I	Diarrhea	-	*C. jejuni*
16	8	F	O	Diarrhea, fever, abdominal cramp	-	*C. jejuni*
17	4	M	O	Diarrhea	-	*C. jejuni*
18	1	M	O	Diarrhea	-	*C. jejuni*
19	44	F	I	Diarrhea	CLL ^5^	*C. jejuni*
20	6 m ^1^	F	O	Diarrhea	Bartter syndrome	*C. jejuni*
21	26	F	O	Diarrhea	-	*C. coli*
22	26	M	O	Diarrhea, abdominal cramp	-	*C. coli*
23	1	M	I	Diarrhea, fever	-	*C. jejuni*
24	3	F	O	Bloody diarrhea, abdominal cramp	-	*C. coli*
25	57	F	O	Diarrhea	Kidney transplantation	*C. jejuni*
26	22	F	O	Diarrhea	-	*C. jejuni*
27	23	F	O	Diarrhea, fever	-	*C. jejuni*
28	59	M	O	Diarrhea, abdominal cramp	Myeloma	*C. jejuni*
29	29	M	O	Diarrhea, fever	-	*C. coli*
30	7	F	O	Diarrhea	-	*C. jejuni*
31	61	F	O	Bloody diarrhea, abdominal cramp, vomiting	Cholecystectomy, diabetes, hypertension, cardiac dysrhythmia, hypothyroidy	*C. concisus*

^1^ m: months; ^2^ F: Female, M: Male; ^3^ O: Outpatient, I: Inpatient; ^4^ IBS: Inflammatory bowel syndrome; ^5^ CLL: Chronic lymphocytic leukemia.

**Table 6:** Antibiotic resistance profiles of the *Campylobacter* isolates [n (%)].

	Ciprofloxacin	Tetracycline	Erythromycin
*C. jejuni* (n: 21)	19	13	1
*C. coli* (n: 9)	9	3	0
*C. concisus* (n: 1)	1	1	0
Total (n: 31)	29 (93.5)	17 (54.8)	1 (3.2)

## Data Availability

The data are included in the article by excluding personal data that may not comply with GDPR regulations.
